# White matter microstructural alterations in patients with neuropathic pain after spinal cord injury: a diffusion tensor imaging study

**DOI:** 10.3389/fneur.2023.1241658

**Published:** 2023-08-24

**Authors:** Dong Dong, Koichi Hosomi, Nobuhiko Mori, Yoshi-ichiro Kamijo, Yohei Furotani, Daisuke Yamagami, Yu-ichiro Ohnishi, Yoshiyuki Watanabe, Takeshi Nakamura, Fumihiro Tajima, Haruhiko Kishima, Youichi Saitoh

**Affiliations:** ^1^Department of Neurosurgery, Graduate School of Medicine, Osaka University, Suita, Osaka, Japan; ^2^Department of Mechanical Science and Bioengineering, Graduate School of Engineering Science, Osaka University, Toyonaka, Osaka, Japan; ^3^Department of Rehabilitation Medicine, Wakayama Medical University, Wakayama, Japan; ^4^Department of Rehabilitation Medicine, Saitama Medical Center, Dokkyo Medical University, Mibu, Tochigi, Japan; ^5^Department of Rehabilitation Medicine, Graduate School of Medicine, Yokohama City University, Yokohama, Kanagawa, Japan; ^6^Department of Rehabilitation Medicine, Kanagawa Rehabilitation Hospital, Atsugi, Kanagawa, Japan; ^7^Department of Neurosurgery, Osaka Gyoumeikan Hospital, Osaka, Japan; ^8^Department of Radiology, Shiga University of Medical Science, Otsu, Shiga, Japan; ^9^Tokuyukai Rehabilitation Clinic, Toyonaka, Osaka, Japan

**Keywords:** neuropathic pain, spinal cord injury, diffusion tensor imaging, white matter microstructural alterations, corpus callosum

## Abstract

**Background:**

Through contrastive analysis, we aimed to identify the white matter brain regions that show microstructural changes in patients with neuropathic pain (NP) after spinal cord injury (SCI).

**Methods:**

We categorized patients with SCI into NP (*n* = 30) and non-NP (*n* = 15) groups. We extracted diffusion tensor maps of fractional anisotropy (FA) and mean (MD), axial (AD), and radial (RD) diffusivity. A randomization-based method in tract-based spatial statistics was used to perform voxel-wise group comparisons among the FA, MD, AD, and RD for nonparametric permutation tests.

**Results:**

Atlas-based analysis located significantly different regions (*p* < 0.05) in the appointed brain atlas. Compared to the non-NP group, the NP group showed higher FA in the posterior body and splenium of the corpus callosum and higher AD in the corpus callosum, internal capsule, corona radiata, posterior thalamic radiation, sagittal stratum, external capsule, cingulum, fornix/stria terminalis, superior longitudinal fasciculus, and uncinate fasciculus.

**Conclusion:**

The results demonstrated that compared with the non-NP group, NP pathogenesis after SCI was potentially related to higher values in FA that are associated with microstructural changes in the posterior body and splenium of the corpus callosum, which could be regarded as central sensitization or network hyperexcitability.

## Introduction

Neuropathic pain (NP) is caused by a lesion or disease of the somatosensory nervous system (1). Causes include spinal cord injury, stroke, phantom limb, diabetic neuropathy, herpes zoster infection, and radiculopathy. Characteristic symptoms of NP are spontaneous continuous pain, shooting pain, allodynia, and hyperalgesia. Spinal cord injury (SCI) is a neurological disorder that severely affects the quality of life of patients and causes NP in more than 50% of them (2–5). Although several therapeutic strategies aim to address NP, it is often not relieved, posing a challenge for pain management (6–8). Therefore, a better understanding of the NP mechanisms is required.

Reorganization of the brain has been reported in patients with NP (9–11). The side contralateral to the symptomatic side in patients with chronic post-traumatic NP showed a decrease in thalamic blood flow (9). Functional magnetic resonance imaging (fMRI) studies have demonstrated that NP could be related to disruption of the default mode network and reorganization of the primary somatosensory cortex (12, 13). Up to 50% of the patients with complete SCI experiencing NP have some activity in the somatosensory cortex but respond poorly to brush stimulation (14). Also, NP has been associated with brain structural changes, especially in white matter (WM) of the central nervous system (10, 15). Diffusion-weighted imaging (DWI), as a revolutionized imaging technique, measures the diffusion of water molecules in tissues. Diffusion tensor imaging (DTI) scrutinizes the three-dimensional shape of the diffusion and provides quantitative information on tissue microstructures (16), that extensively used to measure changes in WM. Yoon et al. (17) used fluorodeoxyglucose-positron emission tomography, T1-anatomical magnetic resonance imaging (MRI), and DTI to examine patients with NP after SCI. Based on whole brain voxel-wise comparison, DTI analysis revealed decreased mean diffusivity in the right internal capsule, cerebral peduncle, prefrontal cortex, and pre-and post-central WM. Additionally, several DTI studies found that patients with NP exhibited significant anatomical changes in the amygdala, thalamus, insula, prefrontal cortex, and posterior parietal lobule that were correlated with the sensory perception of pain (7, 18, 19).

NP is accompanied by changes in brain mechanisms and could be associated with pain perception and threshold (7, 18–20). However, most previous DWI/DTI studies have invariably compared patients with SCI and NP to healthy controls; or included a relatively small NP patient sample of approximately 10 subjects (7, 17, 21). Furthermore, these studies have ignored the differences in the cerebral WM between groups, such as patients with complete and incomplete SCI or cervical and other SCI (17, 21). To the best of our knowledge, only two DTI studies have reported significant results in patients with NP after SCI. Gustin et al. (7) compared healthy controls and patients with or without NP after SCI. They found that patients with NP exhibited higher MD values in the right posterior parietal cortex, right dorsolateral prefrontal cortex, left anterior insula, medial orbitofrontal cortex, and premotor cortex and lower MD values in the ventral pons extending into the ventral midbrain, left amygdala, and right ventroposterior thalamus than patients without NP. Another study (17) compared healthy controls and patients with NP after SCI and found lower MD values in the anterior corona radiata, anterior to posterior limb, and cerebral peduncle in patients with NP than in the healthy controls. However, these studies had limitations such as inadequate number of assessed variables and a small sample size; Gustin et al. (7) recruited 12 and Yoon et al. (17) recruited 10 patients with NP after SCI. Moreover, most studies compared patients with NP after SCI with healthy controls; hence, the results might be more relevant to SCI related changes than to NP. Therefore, their results included a limited number of participant or suffer from a paucity of information regarding FA data.

Therefore, it is necessary to elucidate the mechanism of cerebral WM microstructural changes in patients with and without NP after SCI. Additionally, the changes in brain WM should be investigated for subgroup comparisons, such as NP patients with complete or incomplete SCI. Past studies have mostly focused on brain regions that are directly associated with the pain matrix (2, 6, 22, 23). We consider that different types of SCI, such as complete or incomplete SCI, may influence the microstructural changes of neuropathic pain in brain WM. Additionally, the comparison between NP and non-NP SCI patients has not been thoroughly examined.

This study aimed to locate the brain regions that showed microstructural changes in the WM of patients with NP and to investigate the neural mechanisms underlying NP in patients with SCI, using tract based spatial statistics (TBSS). Furthermore, we sought to evaluate the contribution of different classes of SCI to brain WM tissue alterations. Unlike most previous studies, we compared the DTI data between patients with and without NP after SCI. In addition, the TBSS was used for a whole brain analysis. This could determine the location of the WM microstructural anomalies that are related to and affect NP after SCI.

## Materials and methods

### Patient recruitment and assessment

Fifty patients with SCI, sustained at least 6 months before recruitment, were included in this study. All patients were over 18 years old and had sensory disturbances. As this study aimed to investigate the neural mechanisms in SCI patients with and without NP, healthy subjects were not included.

We excluded patients with the following conditions: dementia, severe aphasia or higher brain dysfunction, major psychiatric disorder, pregnancy, inability to complete the questionnaires, inability to provide consent, and considered ineligible for enrolling in the study by the principal investigator or sub-investigator. This was a multi-center study involving three study centers (Department of Neurosurgery at Osaka University Hospital, Department of Rehabilitation at Wakayama Medical University Hospital, and Department of Rehabilitation at Yokohama City University Hospital) to recruit more study subjects. The patients followed up at the study centers were recruited, a comprehensive patient overview detailed in Supplementary Table S1.

All patients were assessed by the American Spinal Injury Association scale (ASIA) and neurological level of injury (NLI) based on the International Standards for Neurological Classification of SCI (ISNCSCI), Beck Depression Inventory-II (BDI-II), hospital anxiety and depression scale (HADS), functional independence measure (FIM), EuroQOL 5-Dimension 5-Level (EQ-5D-5L), and Japanese adult reading test (JART50). Patients with NP were also examined using the visual analog scale (VAS) of pain intensity, numeric rating scale (NRS) of pain intensity, pain disability assessment scale (PDAS), painDETECT, short-form McGill pain questionnaire 2 (SF-MPQ2), and pain catastrophizing scale (PCS). Qualified clinicians at each center performed all assessments within a month of performing the MRI (24–29).

The DTIs of five patients were excluded from the overall dataset due to quality issues (see the image preprocessing section for details). NP definitions were based on International Association for the Study of Pain (IASP) terminology (1). Subsequently, 45 patients with SCI were investigated, of which 30 had NP (NP group, 26 men and four women), and 15 had no NP (non-NP group, 14 men and one woman). There were three patients with at-level NP (i.e., pain at the level of injury), 11 patients with below-level NP (i.e., pain below the level of injury), and 21 patients with at-and below-level NP.

This study followed the Declaration of Helsinki and the Ethical Guidelines for Medical and Health Research Involving Human Subjects enacted by the Japanese Ministry of Health, Labour and Welfare. The study protocol was approved by the Institutional Review Board of each center (approval numbers: 18079, 2428, and B181200028), and written informed consent was obtained from all participants. Data collection initiated in December 2018 and was completed in September 2020.

### MRI data acquisition

T1-weighted MRI and diffusion-weighted MRI scans were performed using 3 T scanners (Discovery MR750(w), General Electric, Boston, MA, United States; Achieva dStream 3.0 T, Philips, Amsterdam, the Netherlands). T1-weighted images were acquired with the following parameters: repetition time/echo time (TR/TE), 6.9/3.3 ms; flip angle, 10°; field of view, 260× 260 mm; matrix, 256 × 256; slice thickness, 1.2 mm; 156 slices. The DWI were acquired using an Echo Planar imaging sequence (one image with b = 0 s/mm^2^ and 15 motion probing gradient (MPG) directions with b = 1,000 s/mm^2^ for shortening the time and keeping the conditions same at the three departments; slice thickness = 2.6 mm; flip angle = 90°; mean echo time ≈ 67.7 ms; repetition time = 12,000 ms; matrix (reconstruction) = 256 × 256, matrix (acquisition) = 128 × 128; field of view = 156 × 240 × 240 mm; voxel size = 2.6 × 0.94 × 0.94 mm, total acquisition time = 215 s).

### Image preprocessing

DTIprep 1.2.8^1^ was used for quality checks before preprocessing the DWI data. The data of any patient with more than two poverty tensor computations or excessive head movement gradients were excluded from the overall image analysis. After the quality check, the images were converted from the Digital Imaging and Communications in Medicine (DICOM) format to Neuroimaging Informatics Technology Initiative (NIfTI) format using the dcm2nii toolbox.^2^ Diffusion data preprocessing was then performed using FMRIB’s Diffusion Toolbox, a part of the FMRIB Software Library (FSL 6.0.5; www.fmrib.ox.ac.uk/fsl). The preprocessing steps were as follows: (1) Extraction of the brain data from the b = 0 image and T1-weighted image using “bet.” (2) Simultaneous correction of head movement and distortion using “eddy.” The distortion correction handled by non-linearly registering with T1-weighted image. (3) Fitting diffusion tensors on eddy-corrected data to acquire maps of fractional anisotropy (FA) and mean (MD), axial (AD), and radial (RD) diffusivity using “dtifit.”

### Tract-based spatial statistic

TBSS is used to interpret multi-subject diffusion imaging sensitively and objectively.^3^ In the TBSS analysis, we projected FA, MD, AD, and RD data to the mean FA skeleton for more precisely representing the main fiber tracts (WM microstructures). The biological microstructure details that are related to the changes in FA, MD, AD, and RD can help us gain a better understanding of TBSS analysis result. However, this topic is beyond the scope of this paper and we discussed it in Supplementary material.

The TBSS of the FSL was used for voxel-wise statistical analysis of the FA data, after the data preprocessing and FA maps was nonlinearly registered to the MNI standard space using “FNIRT.” The mean FA maps and skeleton were built; the mean FA skeleton had a threshold of 0.2 to represent the main fiber tracts only. The aligned FA, MD, AD, and RD data of the patients were then projected onto the mean FA skeleton. Subsequently, voxel-wise statistical processing was performed on the projected and skeletonized data.

### Statistical analysis

We used a randomization-based method to perform voxel-wise group comparisons and determine the correlations analysis for FA, MD, AD, and RD. The analysis matrix was constructed using the general linear model (GLM) framework, and the nonparametric permutation test was carried out with FSL’s randomization tool, which generated 5,000 permutations. Age was included as a covariate in our study. Additionally, the type of scanner was also included as a covariate in the sensitivity analysis to strengthen robustness of the results. The analysis was performed using the threshold-free cluster enhancement settings^4^ with a statistical significance set at *p* < 0.05 after correcting for family-wise error (FWE). Furthermore, MD, AD, and RD values were compared by the same GLM design, only if significant differences in the FA values were observed. First, we compared the NP and non-NP groups using the above nonparametric permutation test. Second, the same test was also conducted for other intergroup comparisons and correlation analyses. The other intergroup comparisons included complete vs. incomplete SCI, cervical vs. other SCI, FIM normal function vs. dysfunction, and disabling vs. non-disabling pain (NP group). Severity of SCI (complete or incomplete) and levels of SCI (cervical or other) were classified based on the ISNCSCI, then confirmed by vertebral body level of the SCI in MRI or CT images. FIM normal function and dysfunction were categorized as follows; FIM ≥ 90 indicated normal function, and FIM < 90 indicated dysfunction. VAS score ≥ 40 was considered disabling pain and VAS score < 40 was considered non-disabling pain (30). Correlations between the DTI values, such as FA, MD, AD, and RD, and the following patient backgrounds were investigated, the ASIA motor/sensory scores, NLI, EQ-5D-5L, BDI-II, HADS, FIM, and JART50. The scores of VAS, NRS, SF-MPQ2, painDETECT, PDAS, and PCS were analyzed only in the NP group. The “autoaq” tool of the FSL was used for atlas-based analysis to locate the regions of significant differences in the International Consortium of Brain Mapping (ICBM) DTI-81 white matter label atlas^5^ (31). The patients in this study had no lesions within the atlas scope.

## Results

### Demographics

Table 1 shows the demographic and clinical data of patients in the NP and non-NP groups. Patients in the NP group were older (*p* = 0.04) and had higher BDI-II (*p* = 0.04), HADS anxiety (*p* = 0.03), and HADS depression (*p* = 0.02) scores than those in the non-NP group. In addition, the NP group had statistically significantly higher number of patients with incomplete SCI (*p* = 0.02) than the non-NP group, which is common in SCI studies (32). There was a statistically significant difference in the ASIA impairment scale: A~E among NP and non-NP groups (*p* = 0.01). The groups were similar in other patient characteristics.

**Table 1:** Patient characteristics.

Characteristics		NP group (*N* = 30)		Non-NP group (*N* = 15)		*p-*value
		Mean (range) or *N*	SD or *n*%	Mean (range) or *N*	SD or *n*%	
Age (years)		53.4 (37–81)	15.6	42.4 (26–72)	17.8	0.04
Sex (male)		26	87%	14	93%	0.50
Months since injury		132 (6–427)	125	137 (7–569)	174	0.93
Incomplete SCI		17	57%	3	20%	0.03
AIS:^a^	A	13	43%	12	80%	0.01
	B	1	3%	1	7%	
	C	1	3%	0	0%	
	D	15	50%	1	7%	
	E	0	0%	1	7%	
Cervical level of injury		18	60%	6	40%	0.21
NLI^b^		C7	C4~T5	T2	C7 ~ T8	0.31
ASIA motor score (0–100)		56.0 (11–97)	20.2	48.9 (20–100)	20.0	0.27
ASIA sensory score (0–224)		120.1 (24–224)	40.5	114.6 (42–224)	45.9	0.68
Wheelchair dependency		22	73%	13	87%	0.31
EQ-5D-5L score (0–1)		0.59 (0.16–0.89)	0.23	0.71 (0.34–1)	0.22	0.09
BDI-II score (0–63)		14.9 (3–35)	7.6	9.9 (0–23)	7.1	0.04
HADS anxiety score (0–21)		5.5 (0–12)	3.4	3.2 (0–10)	3.3	0.03
HADS depression score (0–21)		6.7 (1–14)	3.5	3.9 (0–10)	3.7	0.02
FIM score (18–126)		102.3 (57–126)	19.7	108.1 (67–126)	14.7	0.32
JART50 score (0–124.1)		101.3 (80.7–117.4)	9.4	97.5 (80.7–124.1)	11.1	0.24
VAS score (0–100 mm)		52.1 (6–100)	24.3			
NRS score (0–10)		4.7 (1–10)	2.3			
SF-MPQ2 score (0–220)		52.5 (4–171)	42.5			
painDETECT score (0–38)		13.3 (1–31)	7.9			
PDAS score (0–60)		23.4 (0–27)	15.5			
PCS score (0–52)		26.4 (3–20)	10.1			

^a^In AIS: A~E, *p*-value is the result of 2 (neuropathic pain and non-neuropathic pain group) × 5 (AIS: A~E) fisher exact test. ^b^In NLI, Mean (range) or *N* is regarded as median, and SD or *n*% is regarded as interquartile range. NP, Neuropathic pain; AIS, ASIA impairment scale; ASIA, American Spinal Injury Association; NLI, Neurological level of injury, EQ-5D-5L, EuroQOL 5 Dimensions 5-Level; BDI-II, Beck depression inventory; HADS, Hospital anxiety and depression scale; FIM, Functional independence measure; JART50, Japanese adult reading test; VAS, Visual analog scale; NRS, Numeric rating scale; SF-MPQ2, short-form McGill Pain Questionnaire 2; PDAS: Pain disability assessment scale; PCS, Pain catastrophizing scale; SCI, Spinal cord injury; SD, Standard deviation.

### DTI analyses

**Figure 1:**
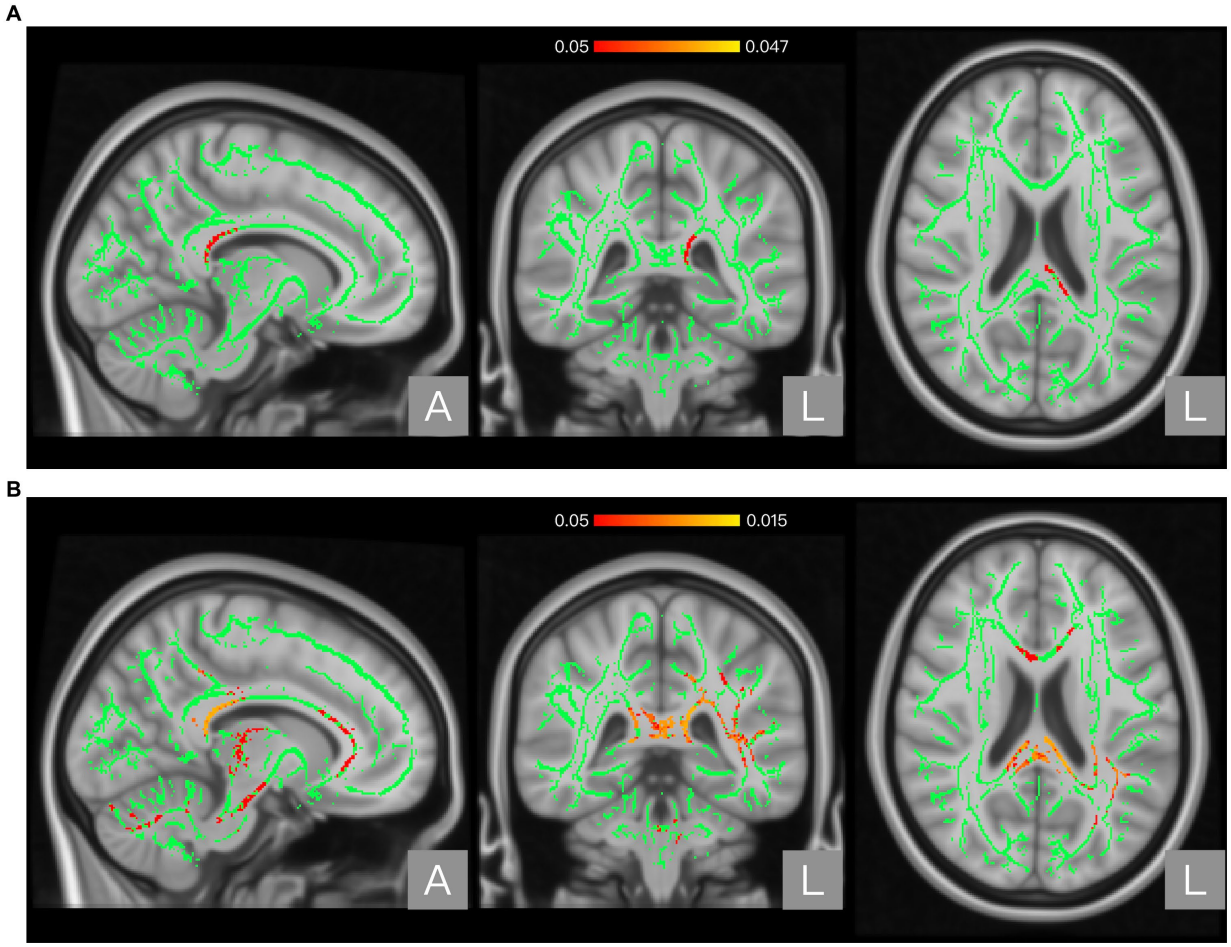
Regions showing significant differences between the neuropathic pain (NP) and non-NP groups in Fractional anisotropy (FA). Panel **(A)** shows the results with age as a covariate. Panel **(B)** shows the results with both age and scanner variations as covariates. The background brain image is the MNI152 standard-space T1-weighted average structural template image (MNI152_T1_1 mm). The green area shows the mean WM skeleton of all patients, using a threshold of 0.2 to represent only the main fiber tracts. The red/yellow area represents the regions with higher FA in patients with NP (at *p* < 0.05 with family-wise error correction and using the threshold-free cluster enhancement settings) when compared with non-NP patients.

**Figure 2:**
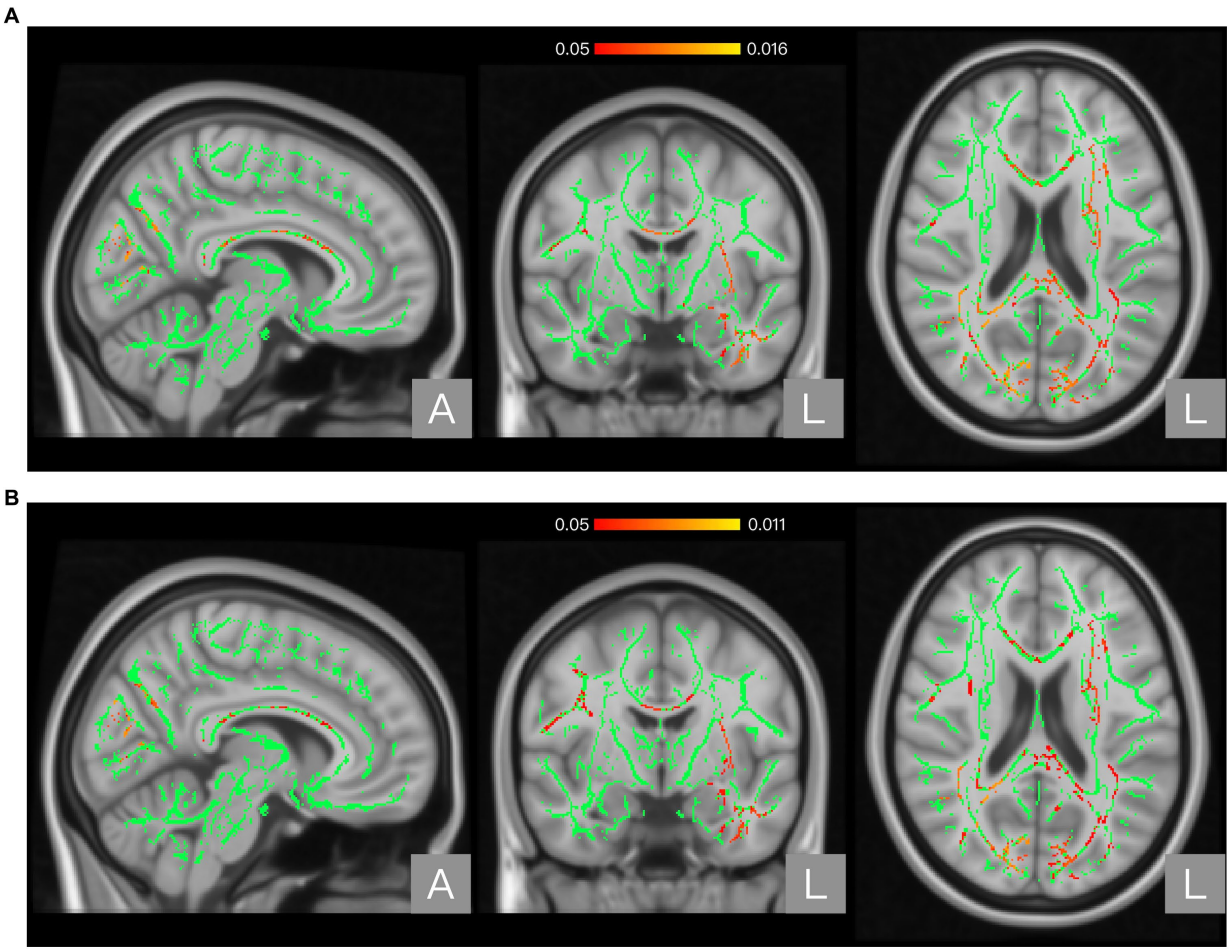
Regions showing significant differences between the neuropathic pain (NP) and non-NP groups in Axial diffusivity (AD). Panel **(A)** shows the results with age as a covariate. Panel **(B)** shows the results with both age and scanner variations as covariates. The background brain image is the MNI152 standard-space T1-weighted average structural template image (MNI152_T1_1 mm). The green area shows the mean WM skeleton of all patients, using a threshold of 0.2 to represent only the main fiber tracts. The red/yellow area represents the regions with higher AD in patients with NP (at *p* < 0.05 with family-wise error correction and using the threshold-free cluster enhancement settings) when compared with non-NP patients.

The global FA, MD, AD, and RD values of the NP and non-NP groups are shown in Table 2. Nonparametric permutation test results showed that the NP group had significantly higher FA in one cluster (*p* < 0.05, FWE corrected) and AD values in three clusters (*p* < 0.05, FWE corrected) than the non-NP group (Table 3). FA in patients with NP was higher in the posterior body and splenium of the corpus callosum (*p* < 0.05, FWE corrected; Figure 1A; Table 3), and AD was higher in the corpus callosum, internal capsule, corona radiata, posterior thalamic radiation, sagittal stratum, external capsule, cingulum (cingulate gyrus), fornix and stria terminalis, superior longitudinal fasciculus, and uncinate fasciculus (*p* < 0.05, FWE corrected; Figure 2A; Table 3) than those without NP. The sensitivity analysis including both age and scanner as covariates at a significance level of *p* < 0.05 (FWE corrected) showed more significant clusters in the FA map than the analysis without the covariate of scanner. Upon elevating the significance level to *p* < 0.03 (FWE corrected), the significant region in the FA map was concentrated within the posterior body and splenium of the corpus callosum (Figure 1B). The sensitivity analysis of the AD map with covariates of both age and scanner revealed similar results to the analysis without the covariate of scanner (Figure 2B). There were no differences in intergroup comparisons, including gender, complete vs. incomplete SCI, cervical vs. other SCI, FIM normal function vs. dysfunction, and disabling vs. non-disabling pain.

**Table 2:** Global FA, MD, AD, and RD values of patients in the NP and non-NP groups.

Characteristic	NP group (*N* = 30)		Non-NP group (*N* = 15)	
	Mean	SD	Mean	SD
Global FA values (×10^−1^)	4.56	1.73	4.49	1.73
Global MD values (×10^−4^)	7.85	3.11	7.83	3.01
Global AD values (×10^−3^)	1.20	0.42	1.19	0.41
Global RD values (×10^−4^)	5.77	3.07	5.80	2.98

NP, Neuropathic pain; FA, Fractional anisotropy; MD, Mean diffusivity; AD, Axial diffusivity; RD, Radial diffusivity; SD, Standard deviation.

**Table 3:** Regions of the clusters showing significantly higher (positive) FA and AD values in the NP group than in the non-NP group.

	Clusters	Voxels	Peak *p*-values (maximum)	Peak coordinates(X, Y, Z in mm)	Regions (in WM tracts)
FA	Cluster 1	306	0.047 ^FWE^	−14, −40, 21	BCC, SCC
AD	Cluster 1	19,669	0.016 ^FWE^	18, −55, 48	GCC, BCC, SCC, ALIC L, PLIC L, RPIC L, ACR L, SCR L R, PCR L R, PTR L R, SS L R, EC L, CCG L, F/ST L, SLF L R, UF L
	Cluster 2	291	0.042 ^FWE^	44, −1, 30	SLF R
	Cluster 3	64	0.049 ^FWE^	−17, −10, 1	PLIC L

^FWE^, *p*-values are corrected for family-wise error, covariate was age. FA, fractional anisotropy; AD, axial diffusivity; NP, Neuropathic pain; WM, White matter; L, Significance in the left; R, Significance in the right; GCC, Genu of corpus callosum; BCC, Body of corpus callosum; SCC, Splenium of corpus callosum; ALIC, Anterior limb of internal capsule; PLIC, Posterior limb of internal capsule; RPIC, Retrolenticular part of internal capsule; ACR, Anterior corona radiata; SCR, Superior corona radiata; PCR, Posterior corona radiata; PTR, Posterior thalamic radiation (including optic radiation); SS, Sagittal stratum (including the inferior longitudinal fasciculus and inferior fronto-occipital fasciculus); EC, External capsule; CCG, Cingulum (cingulate gyrus); F/ST, Fornix (cres)/Stria terminalis (cannot be resolved with current resolution); SLF, Superior longitudinal fasciculus; UF, Uncinate fasciculus.

Following the comparison between the NP and non-NP groups, we conducted a correlation analysis on the regions with significant differences (the posterior body and splenium of the corpus callosum). This analysis was in relation to clinical scores that were significantly different between the NP and non-NP groups, namely BDI-II, HADS anxiety, and HADS depression scores (Table 1). No significant correlations were found with BDI-II. HADS anxiety score demonstrated a positive correlation in FA (*p* = 0.035, peak value with FWE corrected) and a negative correlation in AD (*p* = 0.033). HADS depression score also demonstrated a positive correlation in FA (*p* = 0.049) and no significant correlation in AD (*p* = 0.212). We found no significant correlations in MD and RD.

The significant patient characteristics and clinical scores in the whole brain WM correlation analysis of FA, MD, AD, and RD are shown in Table 4. We found significant positive correlations between FA and the scores of PDAS (NP group), ASIA motor, and JART50, and significant negative correlations with BDI-II scores (NP group), NLI (NP group), and age (*p* < 0.05, FWE corrected). The results of MD and RD showed positive correlations with age and negative correlations with PDAS score and JART50. RD was positively correlated with BDI-II score, and AD was positively correlated with JART50 score and negatively correlated with NLI score and age (see Table 4 for the correlations between the DTI parameters and clinical characteristics; see Supplementary Table S2 for the results of atlas-based analysis).

**Table 4:** Correlations between DTI parameters and clinical characteristics of patients.

	FA		MD		AD		RD	
Characteristics	Positive	Negative	Positive	Negative	Positive	Negative	Positive	Negative
PDAS^a^	*			*				*
ASIA motor	**							
JART50	**			*	*			**
BDI-II^a^		*					*	
NLI^a^		*				**		
Age		***	***			*	***	

^a^In patients with neuropathic pain after spinal cord injury. *p < 0.05, **p < 0.01, ***p < 0.001; family-wise error; “p” means the peak *p*-values (maximum). The NLI was substituted with numbers that could define the injury locations as high and low. For example, C1 was substituted with 1, T1 was substituted with 9, and so on. The details of atlas-based analysis results were shown in Supplementary Tables S2A–S2D. DTI, diffusion tensor imaging; PDAS, Pain disability assessment scale; ASIA motor, American Spinal Injury Association motor sub-score; JART50, Japanese adult reading test; BDI-II, Beck depression inventory; NLI, Neurological level of injury; FA, Fractional anisotropy; MD, Mean diffusivity; AD, Axial diffusivity; RD, Radial diffusivity.

## Discussion

This study investigated microstructural differences in brain WM tracts between patients with and without NP after SCI by analyzing their DTI data. The TBSS results showed significant differences between the NP and non-NP groups. In particular, the NP group exhibited significantly higher FA in the posterior body and splenium of the corpus callosum and higher AD in several brain WM tracts than the non-NP group. Comparisons of complete SCI vs. incomplete SCI, cervical SCI vs. other SCI, FIM normal function vs. dysfunction, and disabling pain vs. non-disabling pain showed no significant differences. The results of correlation analysis have been discussed in Supplementary material.

An SCI affects the afferent and efferent pathways of both sensory and motor tracts and leads to structural and functional alterations in the brain (4, 33). Few DTI studies in patients with NP after SCI have been conducted. Hence, the mechanisms of NP development mechanism and the microstructural differences in certain WM brain regions in patients with NP after SCI have not been determined. In this study, we compared 30 patients with NP with 15 patients without NP after SCI. TBSS analysis revealed microstructural variations; patients with NP had higher FA in the posterior body and splenium of the corpus callosum than patients without NP. Meanwhile, higher AD was observed in several brain WM tracts, including body and splenium of the corpus callosum. Higher FA and AD in the same WM tracts enhance the credibility of the results (34–36). We believe this is the first study to provide evidence based on both FA and AD on the relationship between the specific corpus callosum regions and NP by comparing patients with and without NP having SCI. The corpus callosum is the largest WM commissural bundle in the brain. It connects and processes communication between homologous brain systems in the two hemispheres. It is also associated with the nociceptive threshold and evokes the critical dimensions of the pain experience (37). Fibers form the posterior body of the corpus callosum connecting the parietal lobes (38, 39), fibers of the anterior part of the splenium of the corpus callosum principally connect the parietal and temporal lobes, and fibers of the posterior part connect the occipital lobes (10, 38, 40). Regions of the parietal and temporal lobes are generally related to sensations such as pain and other somatosensory stimuli, space recognition, memory, and emotion. Regions of the parietal lobe connect to the posterior body and anterior splenium of the corpus callosum and function in processing somatosensory information (22). The posterior body and anterior splenium of the corpus callosum are exactly in the regions of our findings.

Cingulate cortex is located immediately above the corpus callosum and plays an important role in the central executive network of pain matrix (41). Posterior cingulate cortex transmits somatosensory information and relays communication with anterior cingulate cortex. Meanwhile, posterior cingulate cortex is connected to the splenium of the corpus callosum. Previous NP and SCI studies (2, 42–45) have confirmed the direct or indirect association of the corpus callosum with chronic pain. Consistent with previous studies, it is posited that NP could be related to disruption of the default mode network (12, 13). Additionally, the posterior cingulate cortices of both sides are connected structurally and functionally by the posterior segment of the corpus callosum, which is a key functional hub of the default mode network (22, 40, 42, 45–47). Torrecillas-Martínez et al. (20) used DTI to assess brain WM changes related to subjective pain perception in an acute surgical pain model. They compared three postsurgical pain stages and showed significant positive associations between FA in the posterior parts of the corpus callosum, corticospinal tract, and corona radiata and pain processing (20). NP usually arises from central sensitization and nociceptive system hyperexcitability (11, 48). This NP mechanism could be employed for its management by directly suppressing network excitatory activity and thus cortical hyperexcitability or by enhancing its inhibition. Our study results showed WM microstructural differences in the posterior body and splenium of the corpus callosum between patients with and without NP after SCI. As this result was obtained for both FA and AD, we may strongly suggest that these differences occur in the WM and may be caused by increased density of axonal packing or excessive myelination. This might be related to disturbed somatosensory information processing or interhemispheric communication that generated excitatory activity and led to cortical hyperexcitability associated with NP perception.

Although TBSS has strong localization capability in comparative analysis, the statistical power still may have some limitations (49). To overcome this, we used threshold-free cluster enhancement method, which is more stable and circumspect than traditional cluster-based thresholding method (50). However, due to the limitations of atlas-based correlation analysis, we believe that locations of atlas-based analysis results (Supplementary Tables S2A–S2D) are not reliable enough and can only be used as a reference in this study, and discussed in Supplementary material.

This study had several limitations. First, due to the need to standardize the collection criteria of the study population, data collection for this study took a considerable amount of time, resulting in certain limitations to imaging protocols. Specifically, Since the subjects were all patients, according to the clinical perspective, we took DWI in 15 MPG directions in order to make the scanning time short enough. 15 directions satisfy the conditions of TBSS analysis which need more than 6 directions, (see text footnote 3) but still probably a relatively low number of directions in DTI studies. Second, although we attempted to unified imaging protocols and incorporated the scanner difference as a covariate to minimize scanner effects, it is possible that data harmonization approaches could further improve the removal of scanner effects. For future research, it may be beneficial to consider incorporating methods such as imaging phantom acquisitions into study design. Third, our study included a larger number of patients with SCI, but the population differences between the two groups (NP group, *n* = 30; non-NP group, *n* = 15) were insufficiently large. Because our study started with a fixed number of participants, the number of participants in the two groups was not controlled at the time of recruitment. This could result in the high incidence of NP in patients with SCI (4, 5, 33, 51). To minimize the potential impact of these confounding factors on the results, we implemented a GLM design and employed a relatively stringent threshold-free cluster enhancement setting. Despite our efforts, we recognize that the effect of these factors cannot be entirely dismissed. In addition, even though our study considered enough variables, multivariate analysis of the preprocessed TBSS data could provide more powerful evidence across multiple modalities and variables. However, this feature was not available in the FSL. A multimodal approach for the FSL is under development. Future studies using this approach could provide more evidence in this research area.

## Conclusion

This is the first study to use TBSS and a relatively large sample to provide evidence for the effects of NP/non-NP in patients with SCI based on DTI data. Our results indicated that patients with NP after SCI had more significant WM microstructural changes in the posterior body and splenium of the corpus callosum than those without NP. As NP is associated with central sensitization and network hyperexcitability of the nociceptive system, the observed increases in FA and AD within the splenium of the corpus callosum suggest the possibility of central sensitization or network hyperexcitability, which may impair somatosensory information processing or interhemispheric communication. The present study has contributed to a better understanding of the neural basis of neuropathic pain. These findings may inform the development of more precise and effective treatment strategies for this debilitating condition.

## Data availability statement

The raw data supporting the conclusions of this article will be made available by the authors, without undue reservation.

## Ethics statement

The study protocol was reviewed and approved by the Institutional Review Boards of each center, these are: Ethical Review Board of Osaka University Hospital (approval number: 18079), Research Ethics Committee of Wakayama Medical University (approval number: 2428) and Research Ethics Committee for Medical and Health Research Involving Human Subjects of Yokohama City University Hospital (approval number: B181200028). The studies were conducted in accordance with the local legislation and institutional requirements. The participants provided their written informed consent to participate in this study.

## Author contributions

KH, Y-iK, Y-iO, YW, TN, FT, HK, and YS: conception and design. KH, NM, Y-iK, YF, and DY: acquisition of data. DD and KH: analysis and interpretation of data. DD: drafting the article. DD, KH, NM, Y-iK, YF, DY, Y-iO, YW, TN, FT, HK, and YS: discussing the results and critically reviewing the manuscript. All authors contributed to the article and approved the submitted version.

## Funding

This study was partly supported by the Japan Agency for Medical Research and Development (grants: 20ek0610017, 19ek0610016, 21dm0307007, and 21dm0307008) and the Japan Society for the Promotion of Science KAKENHI (grants: 18K08993, 19K19867, and 22K09206). The funders had no role in study design; the collection, analysis and interpretation of data; writing of this report; or the decision to submit the article for publication.

## Conflict of interest

The authors declare that the research was conducted in the absence of any commercial or financial relationships that could be construed as a potential conflict of interest.

## Publisher’s note

All claims expressed in this article are solely those of the authors and do not necessarily represent those of their affiliated organizations, or those of the publisher, the editors and the reviewers. Any product that may be evaluated in this article, or claim that may be made by its manufacturer, is not guaranteed or endorsed by the publisher.

## Footnotes

^1^https://www.nitrc.org/projects/dtiprep/

^2^https://people.cas.sc.edu/rorden/mricron/index.HTML

^3^https://fsl.fmrib.ox.ac.uk/fsl/fslwiki/TBSS

^4^https://fsl.fmrib.ox.ac.uk/fsl/fslwiki/Randomise/UserGuide

^5^http://www.bmap.ucla.edu/portfolio/atlases/ICBM_DTI-81_Atlas/
