# Supplementary material for: White matter microstructural alterations in patients with neuropathic pain after spinal cord injury: a diffusion tensor imaging study
Source: Front Neurol. 2023 Aug 24;14:1241658. doi: 10.3389/fneur.2023.1241658 (PMC10484711; doi:10.3389/fneur.2023.1241658)
Supplement: Supplementary file 1 [file Data_Sheet_1.pdf]

Supplementary Material

White matter microstructural alterations in patients with neuropathic pain after spinal cord injury: a diffusion tensor imaging study

Dong Dong, Koichi Hosomi*, Nobuhiko Mori, Yoshi-ichiro Kamijo, Yohei Furotani, Daisuke Yamagami, Yu-ichiro Ohnishi, Yoshiyuki Watanabe, Takeshi Nakamura, Fumihiro Tajima, Haruhiko Kishima, Youichi Saitoh

*** Correspondence:** Koichi Hosom: k-hosomi@nsurg.med.osaka-u.ac.jp

Discussion on the diffusion tensor imaging (DTI) metrics

We analyzed the DTI metrics in this study, which included fractional anisotropy (FA), mean diffusivity (MD), axial diffusivity (AD) and radial diffusivity (RD). As the DTI metrics are calculated from different diffusion tensor, the biological microstructure relates to the changes of FA, MD, AD and RD are much different.[1] FA can measure the microstructural integrity, is particularly sensitive to microstructural changes, and is especially important in analyzing anatomic heterogeneity in DTI. Higher values in FA may lead to higher WM, dense axonal packing and myelination, and lower gray matter (GM), axonal degeneration, and demyelination. However, because FA is a “summary” of microstructural integrity, FA is less sensitive to distinguishing the types of changes (2). Therefore, to distinguish the specific types of changes in FA, it is necessary to refer the changes in MD, AD and RD. MD is sensitive to necrosis and edema; however, it can hardly be used to measure the changes in WM and GM. Contrary to FA, higher values in MD may indicate higher axonal degeneration and demyelination, and lower density of axonal packing and myelination. Despite this, AD is more variable than FA, it can be used to measure the WM/GM changes. However, AD is still not sufficient to be used as the indicator of dense axonal packing, myelination and demyelination. Higher values of AD shows higher WM, and lower GM and axonal degeneration. RD is sensitive to myelination and demyelination as well as axonal density and diameters. Contrary to FA and AD, higher values of RD indicate higher GM, axonal degeneration, demyelination, and lower WM, dense axonal packing, and myelination.[1–3]

Discussion on the correlations results

We found that the scores of pain disability assessment scale (PDAS), American spinal injury Association (ASIA) motor, and Japanese adult reading test (JART50) were positively correlated with FA. The Beck depression inventory (BDI)-II scores, neurological level of injury (NLI), and age were negatively correlated with FA. In addition, MD, AD and RD data also correlated with some clinical characteristics.

Our correlation analysis results (Table 4) showed that PDAS scores (neuropathic pain [NP] group)

were positively correlated with FA values, negatively correlated with MD and RD values. Reference to the biological microstructure that relates to the changes of the DTI metrics.[1–3] This means that patients with NP after spinal cord injury (SCI), who have a higher level of pain disability, may have higher dense axonal packing and myelination, and lower axonal degeneration and demyelination. ASIA motor scores were positively correlated with FA values. This means that patients with SCI who exhibit higher ASIA motor may have higher microstructural integrity. JART50 scores which represent intelligence quotient were positively correlated with FA and AD values and negatively correlated with MD and RD values. This means that patients with SCI that exhibit higher JART50 scores may have higher WM, dense axonal packing and myelination, and lower axonal degeneration and demyelination. BDI-II scores (NP group) were negatively correlated with FA values and positively correlated with RD. This means that patients with NP after SCI showed an increased inclination for depression, and this may cause a decrease in dense axonal packing and myelination and increase in axonal degeneration and demyelination. NLI (in the NP group) were negatively correlated with FA and AD values. This may that mean the more caudal the SCI, the lower WM microstructures were exhibited in NP group. Age was negatively correlated with FA and AD values and positively correlated with MD and RD values, suggesting that WM, dense axonal packing, and myelination may be lower; axonal degeneration and demyelination may increase with age in patients with SCI.

References

1. Alexander AL, Hurley SA, Samsonov AA, Adluru N, Hosseinbor AP, Mossahebi P, Tromp do P m., Zakszewski E, Field AS. Characterization of Cerebral White Matter Properties Using Quantitative Magnetic Resonance Imaging Stains. *Brain Connect* (2011) 1:423–446. doi: 10.1089/brain.2011.0071
2. Alexander AL, Lee JE, Lazar M, Field AS. Diffusion Tensor Imaging of the Brain. *Neurotherapeutics* (2007) 4:316–329. doi: 10.1016/j.nurt.2007.05.011
3. Feldman HM, Yeatman JD, Lee ES, Barde LHF, Gaman-Bean S. Diffusion tensor imaging: A review for pediatric researchers and clinicians. *J Dev Behav Pediatr* (2010) 31:346–356. doi: 10.1097/DBP.0b013e3181dcaa8b
4. Feldman HM, Yeatman JD, Lee ES, Barde LHF, Gaman-Bean S, Alexander AL, Lee JE, Lazar M, Field AS, Hurley SA, et al. No Title.

Table S1 Patient overview at each center.

Characteristics	Center 1<br>Mean/N	Center 2<br>Mean/N	Center 3<br>Mean/N
NP patients	5	11	14
Non-NP patients	7	6	2
Age (years)	52.4	55.6	52.2
Sex (male)	10	14	16
Incomplete SCI	6	6	8

Notes: Center 1, Department of Neurosurgery at Osaka University Hospital (Discovery MR750, General Electric, Boston, MA, USA); Center 2, Department of Rehabilitation at Wakayama Medical University Hospital (Achieva dStream 3.0T, Philips, Amsterdam, the Netherlands); Center 3, Department of Rehabilitation at Yokohama City University Hospital (Discovery MR750w, General Electric, Boston, MA, USA); NP, Neuropathic pain.

Table S2A Significance in atlas-based analysis of FA correlations with PDAS score, ASIA motor score, JART 50 score, BDI-II score, NLI, and age.

WM tracts (FA)	Positive correlation			Negative correlation		
	PDAS score[a]	ASIA motor score	JART50 score	BDI-II score[a]	NLI[a]	Age
MCP		S	S	S		
PCT		S	S	S		
GCC	S	S	S	S		S
BCC	S	S	S	S	S	S
SCC	S	S	S	S	S	S
FCBF		S		S		S
CT		L, R	L, R	L		
ML		L, R	L, R	L, R		
ICP		L, R	L, R	L, R		
SCP		L, R	L, R	R		
CP	L	L, R	L, R	L		L, R
ALIC	L	L, R	L, R	L		L, R
PLIC	L	L, R	L, R	L, R	L	L, R
RPIC	L	L, R	L, R	L	L	L, R
ACR	L	L, R	L, R	L, R		L, R
SCR	L	L, R	L, R	L, R	L	L, R
PCR	L	L, R	L, R	L, R	L	L, R
PTR	L	L, R	L, R	L, R	L	L, R
SS	L	L, R	L, R	L, R	L	L, R
EC	L	L, R	L, R	L, R		L, R
CCG	L, R	L, R	L, R	R		L, R
CH		L, R	L, R			L
F/ST	L	L, R	L, R	L	L	L, R
SLF	L	L, R	L, R	L, R	L	L, R
SFOF	L	L, R	L, R			L
UF		L, R	L, R	L		L
TAP		R	R			
**Peak p values (maximum)[FWE]**	0.030	0.005	0.003	0.021	0.041	<0.001

Notes:

[a]: In the NP group.

[FWE]: p values are corrected for family-wise error.

The NLI was substituted with numbers that could define the injury locations as high and low. For example, C1 was substituted with 1, T1 was substituted with 9.

Abbreviations:

S, Significance; L, Significance in the left; R, Significance in the right; WM, White matter; FA, Fractional anisotropy; MD, Mean diffusivity; AD, Axial diffusivity; RD, Radial diffusivity; PDAS, Pain disability assessment scale; ASIA, American Spinal Injury Association; JART50, Japanese adult reading test; BDI-II, Beck depression inventory; NLI, Neurological level of injury; MCP, Middle cerebellar peduncle; PCT, Pontine crossing tract (a part of MCP); GCC, Genu of corpus callosum; BCC, Body of corpus callosum; SCC, Splenium of corpus callosum; FCBF, Fornix (column and body

of fornix); CT, Corticospinal tract; ML, Medial lemniscus; ICP, Inferior cerebellar peduncle; SCP, Superior cerebellar peduncle; CP, Cerebral peduncle; ALIC, Anterior limb of internal capsule; PLIC, Posterior limb of internal capsule; RPIC, Retrolenticular part of internal capsule; ACR, Anterior corona radiata; SCR, Superior corona radiata; PCR, Posterior corona radiata; PTR, Posterior thalamic radiation (including optic radiation); SS, Sagittal stratum (including the inferior longitudinal fasciculus and inferior fronto-occipital fasciculus); EC, External capsule; CCG, Cingulum (cingulate gyrus); CH, Cingulum (hippocampus); F/ST, Fornix (cres)/Stria terminalis (cannot be resolved with current resolution); SLF, Superior longitudinal fasciculus; SFOF, Superior fronto-occipital fasciculus (could be a part of the anterior internal capsule); UF, Uncinate fasciculus; TAP, Tapetum

Table S2B Significance in atlas-based analysis of MD correlations with age, PDAS score, and JART 50 score.

WM tracts (MD)	Positive correlation	Negative correlation	
	Age	PDAS score[a]	JART50 score
MCP			
PCT			
GCC	S	S	S
BCC	S	S	S
SCC	S	S	S
FCBF	S		
CT			
ML			
ICP			
SCP			
CP	L		
ALIC	L, R	L, R	R
PLIC	L, R	L, R	
RPIC	L, R	L	L
ACR	L, R	R	R
SCR	L, R	L, R	L
PCR	L, R	L	L
PTR	L, R	L	L
SS	L, R	L	L
EC	L, R	L, R	R
CCG	L, R	R	L
CH	L		
F/ST	L, R	L	
SLF	L, R	L	L
SFOF	L, R		
UF	L, R		
TAP	R		
**Peak *p* values (maximum)[FWE]**	<0.001	0.023	0.018

Notes:

[a]: In the NP group.

[FWE]: *p* values are corrected for family-wise error.

Abbreviations:

S, Significance; L, Significance in the left; R, Significance in the right; WM, White matter; FA, Fractional anisotropy; MD, Mean diffusivity; AD, Axial diffusivity; RD, Radial diffusivity; PDAS, Pain disability assessment scale; ASIA, American Spinal Injury Association; JART50, Japanese adult reading test; BDI-II, Beck depression inventory; NLI, Neurological level of injury; MCP, Middle cerebellar peduncle; PCT, Pontine crossing tract (a part of MCP); GCC, Genu of corpus callosum; BCC, Body of corpus callosum; SCC, Splenium of corpus callosum; FCBF, Fornix (column and body of fornix); CT, Corticospinal tract; ML, Medial lemniscus; ICP, Inferior cerebellar peduncle; SCP, Superior cerebellar peduncle; CP, Cerebral peduncle; ALIC, Anterior limb of internal capsule; PLIC, Posterior limb of internal capsule; RPIC, Retrolenticular part of internal capsule; ACR, Anterior corona

radiata; SCR, Superior corona radiata; PCR, Posterior corona radiata; PTR, Posterior thalamic radiation (including optic radiation); SS, Sagittal stratum (including the inferior longitudinal fasciculus and inferior fronto-occipital fasciculus); EC, External capsule; CCG, Cingulum (cingulate gyrus); CH, Cingulum (hippocampus); F/ST, Fornix (cres)/Stria terminalis (cannot be resolved with current resolution); SLF, Superior longitudinal fasciculus; SFOF, Superior fronto-occipital fasciculus (could be a part of the anterior internal capsule); UF, Uncinate fasciculus; TAP, Tapetum

Table S2C Significance in atlas-based analysis of AD correlations with JART 50 score, NLI, and age.

WM tracts (AD)	Positive correlation	Negative correlation	
	JART50 score	NLI[a]	Age
MCP		S	
PCT			
GCC	S	S	
BCC	S	S	S
SCC	S	S	S
FCBF			
CT			
ML		R	
ICP			
SCP		R	
CP	L, R	R	
ALIC	L	L, R	
PLIC	L, R	R	L
RPIC	L, R	L, R	L
ACR	L	L	L
SCR	L, R	L, R	L
PCR	R	L, R	L
PTR	L, R	L, R	L
SS	L, R	R	
EC	L	R	L
CCG	L	L, R	
CH			
F/ST	L, R	R	
SLF	R	L, R	L
SFOF	L	R	
UF			
TAP	R	R	
**Peak *p* values (maximum)[FWE]**	0.022	0.007	0.037

Notes:

[a]: In the NP group.

[FWE]: *p* values are corrected for family-wise error.

Abbreviations:

S, Significance; L, Significance in the left; R, Significance in the right; WM, White matter; FA, Fractional anisotropy; MD, Mean diffusivity; AD, Axial diffusivity; RD, Radial diffusivity; PDAS, Pain disability assessment scale; ASIA, American Spinal Injury Association; JART50, Japanese adult reading test; BDI-II, Beck depression inventory; NLI, Neurological level of injury; MCP, Middle cerebellar peduncle; PCT, Pontine crossing tract (a part of MCP); GCC, Genu of corpus callosum; BCC, Body of corpus callosum; SCC, Splenium of corpus callosum; FCBF, Fornix (column and body of fornix); CT, Corticospinal tract; ML, Medial lemniscus; ICP, Inferior cerebellar peduncle; SCP, Superior cerebellar peduncle; CP, Cerebral peduncle; ALIC, Anterior limb of internal capsule; PLIC, Posterior limb of internal capsule; RPIC, Retrolenticular part of internal capsule; ACR, Anterior corona radiata; SCR, Superior corona radiata; PCR, Posterior corona radiata; PTR, Posterior thalamic radiation

(including optic radiation); SS, Sagittal stratum (including the inferior longitudinal fasciculus and inferior fronto-occipital fasciculus); EC, External capsule; CCG, Cingulum (cingulate gyrus); CH, Cingulum (hippocampus); F/ST, Fornix (cres)/Stria terminalis (cannot be resolved with current resolution); SLF, Superior longitudinal fasciculus; SFOF, Superior fronto-occipital fasciculus (could be a part of the anterior internal capsule); UF, Uncinate fasciculus; TAP, Tapetum

Table S2D Significance in atlas-based analysis of RD correlations with BDI-II score, age, PDAS score, and JART 50 score.

WM tracts (RD)	Positive correlation		Negative correlation	
	**BDI-II score[a]**	**Age**	**PDAS score[a]**	**JART50 score**
MCP			S	S
PCT			S	S
GCC		S	S	S
BCC	S	S	S	S
SCC		S	S	S
FCBF		S		
CT			L	L, R
ML				L, R
ICP				L, R
SCP			L	L, R
CP		L	L	L, R
ALIC		L, R	L	L, R
PLIC		L, R	L	L, R
RPIC		L, R	L	L, R
ACR		L, R	L	L, R
SCR		L, R	L	L, R
PCR		L, R	L	L, R
PTR		L, R	L	L, R
SS		L, R	L	L, R
EC		L, R	L	L, R
CCG		L, R	L, R	L, R
CH		L, R		L, R
F/ST		L, R	L	L, R
SLF		L, R	L	L, R
SFOF		L, R		R
UF		L, R		L, R
TAP		R		R
**Peak *p* values (maximum)[FWE]**	0.046	<0.001	0.021	0.009

Notes:

[a]: In the NP group.

[FWE]: *p* values are corrected for family-wise error.

Abbreviations:

S, Significance; L, Significance in the left; R, Significance in the right; WM, White matter; FA, Fractional anisotropy; MD, Mean diffusivity; AD, Axial diffusivity; RD, Radial diffusivity; PDAS, Pain disability assessment scale; ASIA, American Spinal Injury Association; JART50, Japanese adult reading test; BDI-II, Beck depression inventory; NLI, Neurological level of injury; MCP, Middle cerebellar peduncle; PCT, Pontine crossing tract (a part of MCP); GCC, Genu of corpus callosum; BCC, Body of corpus callosum; SCC, Splenium of corpus callosum; FCBF, Fornix (column and body of fornix); CT, Corticospinal tract; ML, Medial lemniscus; ICP, Inferior cerebellar peduncle; SCP, Superior cerebellar peduncle; CP, Cerebral peduncle; ALIC, Anterior limb of internal capsule; PLIC, Posterior limb of internal capsule; RPIC, Retrolenticular part of internal capsule; ACR, Anterior corona

radiata; SCR, Superior corona radiata; PCR, Posterior corona radiata; PTR, Posterior thalamic radiation (including optic radiation); SS, Sagittal stratum (including the inferior longitudinal fasciculus and inferior fronto-occipital fasciculus); EC, External capsule; CCG, Cingulum (cingulate gyrus); CH, Cingulum (hippocampus); F/ST, Fornix (cres)/Stria terminalis (cannot be resolved with current resolution); SLF, Superior longitudinal fasciculus; SFOF, Superior fronto-occipital fasciculus (could be a part of the anterior internal capsule); UF, Uncinate fasciculus; TAP, Tapetum
